# Economic Cost of Ovine Johne’s Disease in Clinically Affected New Zealand Flocks and Benefit-Cost of Vaccination

**DOI:** 10.3390/vetsci5010016

**Published:** 2018-01-29

**Authors:** Milan Gautam, Peter Anderson, Anne Ridler, Peter Wilson, Cord Heuer

**Affiliations:** 1 EpiCentre, School of Veterinary Science, Massey University, Palmerston North 4442, New Zealand; p.r.wilson@massey.ac.nz (P.W.); c.heuer@massey.ac.nz (C.H.); 2 The Vet Centre Marlborough, Blenheim 7201, New Zealand; pa@vetmarlborough.co.nz; 3 School of Veterinary Science, Massey University, Palmerston North 4442, New Zealand; a.l.ridler@massey.ac.nz

**Keywords:** Ovine Johne’s disease, economics, mortality, vaccination, New Zealand

## Abstract

The aims of this study were to estimate the on-fam economic cost of ovine Johne’s disease (OJD) based on collected incidence and mortality data, and the benefit-cost of OJD vaccination in typical OJD affected flocks in New Zealand after having vaccinated for a number of years. Owners of 20 sheep breeding and finishing farms known to be clinically affected by ovine Johne’s disease in New Zealand participated in the study and were monitored for up to two years. Farms were categorized as fine-wool (Merino, Half-Bred, Corriedale, n = 15), and other breeds (Romney, composite breeds, n = 5). Ovine JD was confirmed by gross- and histo-pathology in 358 ewes culled due to chronic progressive wasting. An additional 228 ewes with low body condition score (BCS), but not targeted for culling, were tested with ELISA to estimate the proportion of OJD in ewes in the lower 5% BCS of the flock. Calculations were done separately for fine-wool and other breeds. Based on the data, mortality due to OJD, its associated cost and the benefit-cost of vaccination were evaluated for a hypothetical farm with 2000 ewes by stochastic simulation. Total ewe mortality was similar in fine-wool and other breeds, but the estimated mortality due to OJD was 2.7 times as high in fine-wool (median 1.8%, interquartile range IQR 1.2–2.7%) than other breeds (median 0.69%, IQR 0.3–1.2%), but with large variation between farms. ELISA results demonstrated fine-wool sheep had a higher seroprevalence than other breeds (39%, 95% CI 18–61% vs. 9%, 95% CI 0–22%). Stochastic modelling indicated that the average annual cost of mortality due to OJD in a flock of 2000 ewes was NZD 13,100 (IQR 8900–18,600) in fine-wool and NZD 4300 (IQR 2200–7600) in other breeds. Vaccinating replacement lambs against OJD may be cost-effective in most flocks when the pre-vaccination annual ewe mortality due to OJD is >1%. To make the best-informed decision about vaccination it is therefore essential for farmers to accurately diagnose OJD to establish incidence.

## 1. Introduction

Sheep farming contributes significantly to the New Zealand (NZ) economy. The sheep industry earned NZD 3.7 billion in the year ending in June 2016, primarily through exporting meat (lamb and mutton) and raw wool fibre [1].

Conventionally New Zealand sheep production is entirely pasture-based and characterised by large flocks. Meat breeds such as Romney and composite (mixed) breed sheep comprise more than 50% of the national population while fine-wool breeds such as Merino and Corriedale comprise less than 10% [1]. Generally, the health status of sheep in New Zealand is good although several infectious diseases, including paratuberculosis, are endemic in the country [2].

Paratuberculosis, often synonymously only referred to as Johne’s disease (JD), is a chronic bacterial disease caused by *Mycobacterium avium* subspecies *paratuberculosis* (Map) in ruminants. In this study the term OJD refers to clinical disease, usually resulting in mortality. Map infection is widespread in New Zealand with at least 75% of sheep flocks infected [3]. About 2–6% of flocks were believed to be clinically affected [4] and annual incidence of clinical disease within affected flocks was estimated to be about 1% or lower [5,6]. However, those rates were not supported by longitudinal flock monitoring data, hence no robust estimates of incidence are available. Nevertheless, the incidence and prevalence of Map infection and disease is likely to be economically important on some farms. Affected farms suffer economic losses due to sheep deaths and reduced production [7].

There are limited data on economic cost of ovine JD (OJD) to the New Zealand sheep industry. The only analysis, which was based on a simulation rather than physically monitored production effects, was published almost two decades ago [8]. It assumed that if 6% of flocks were infected the estimated minimum annual cost to the industry was NZD 0.9 million. Alternatively, if 70% of flocks were infected, a scenario that is more representative of the current prevalence [3], the estimated annual loss would have been NZD 9.9 million [8].

While some, albeit limited, data now exist for the production effects of clinical JD on some New Zealand sheep farms [7], there is no well-researched current assessment of the economic cost of OJD or Map infection at farm level. A vaccine is registered in New Zealand to control OJD. However, the benefit-cost of OJD vaccination is unknown. Hence this study aimed to investigate mortality due to OJD and to evaluate the cost of OJD in a typical infected flock of fine-wool or other breeds under pastoral conditions in New Zealand. Based on the results, an additional aim was to estimate the cost-benefit of vaccination.

## 2. Materials and Methods

All manipulations performed on animals were approved by the Massey University Animal Ethics Committee (MUAEC 12/75). Where expert opinion was required, they were those of co-authors P. Anderson and A. Ridler.

### 2.1. Farms and Data Collection

Twenty sheep farms were enrolled in the study comprising fifteen fine-wool (Merino, Half-Bred, and/or Corriedale) and five other (Romney and/or composite) breeds. All fine-wool farms were in the South Island while the other breed farms were in the North Island of New Zealand. A half-bred, classified as a fine-wool breed in this study, is a cross-breed between a Merino ewe and a Romney or English Leicester ram. A composite breed, classified as other breed, is a combination of diverse mutton breeds.

Farms were enrolled between August 2012 and July 2013 and monitored until to June 2014 to obtain tallies of ewes at mating, scanning, set-stocking for lambing, and tailing. They also contributed ewes for necropsy and/or ELISA. Complete tallies were provided from 17 farms (13 fine-wool and 4 other), which had flock sizes range from 785 to 20,104 ewes. Of these 17 farms, 13 were monitored over two years and four over one year, providing ewe count (tally) data from more than 100,000 ewes over a total of 30 farm-years (Table 1). In addition to the ewe tally data, these farms also provided annual tailing data, which represented the number of lambs tailed (at 3–6 weeks of age) per ewe per farm-year. This tailing percentage was used as a proxy for lambing percentage, a parameter used in the economic analyses described later. The remaining three farms did not provide tallies and only contributed ewes for necropsy or ELISA.

### 2.2. Annual Mortality due to OJD

We estimated the annual ewe mortality rate due to OJD separately for each breed-type based on three sequential steps. The first was the overall incidence rate of annual ewe mortality determined from ewe tallies (ewes that died during the year/ewes present at start of the season) at mating in March–May in the year of enrolment, at ultrasound scanning for pregnancy in June–July, at set stocking for lambing in August–September, at tailing/weaning in October–January, and at the next mating in March. Ewes missing or unaccounted for at tallies were assumed to have died. The overall ewe mortality per farm-year was the cumulative number of missing ewes divided by total number of ewes at the first count.

The second step established the proportion of total mortality that was ‘likely OJD related’. It was required to correct for the potential selection bias for necropsy (step 3). The estimate was based on records from the 14 farms from which farmer-diagnosed causes of death were available. Causes were categorised as either ‘likely OJD related’ or ‘likely OJD unrelated’. The ‘likely OJD-related’ category was regarded as a reasonable representation of the ewes submitted to necropsy by farmers and comprised three types of mortality causes: ‘possibly Johne’s’, ‘dog tucker’ (used as dog food) and ‘found dead on the farm’.

The third step was the proportion of likely OJD related ewes (step 2) confirmed to be OJD by post mortem. It was based on necropsy of 358 ewes from 19 farms with a body condition score (BCS) of one on a scale of one to five. These ewes were selected by farmers and necropsied by local veterinarians. Gross pathological findings were confirmed by histology of fixed ileocecal valve and lymph node, distal ileum, terminal jejunum and mesenteric lymph node, and classified as either OJD positive, i.e., conforming to lesion categories one to three described by Pérez et al. [9], or negative.

The overall annual ewe mortality rate due to OJD was calculated for each breed type as = overall incidence rate of annual ewe mortality rate step 1 × proportion of likely OJD related mortality (step 2) × proportion of confirmed OJD mortality step 3.

### 2.3. Proportion of OJD-Affected Ewes

An OJD-affected ewe was defined as one which was presumed would progress to clinical OJD, being of low body condition as identified by farmers at the time of and tested positive by serum ELISA. To estimate the proportion of affected ewes, a sample of 228 ewes (range 2–69 per flock from 15 farms) with BCS ≤ 1.5 were tested by serum ELISA at New Zealand Veterinary Pathology Limited. Ewes tested with ELISA were not targeted for culling per se, but represented ewes with low body condition in the flock. Selection of ewes sampled for ELISA testing was done jointly by farmers and their vets when the latter visited the farm for sampling ewes for post-mortem. Based on expert opinion, we assumed that 5% of ewes of a typical flock in either breed category would have BCS ≤ 1.5. If an ELISA positive ewe was necropsied it was included in the necropsy group. An estimate of the number of OJD-affected ewes in a flock was calculated from the proportion of ewes with BCS ≤ 1.5 that were ELISA positive among the 5% of ewes with low BCS.

Data from OJD-affected ewes were used to estimate pre-clinical loss (poor reproductive performance, lower carcass weight of cull-ewes). The pre-clinical loss was calculated by the difference in productive lifetime among the necropsied ewes (i.e., age at culling/death, which was estimated based on ear marks or ear tags) between OJD-confirmed and non-OJD ewes, multiplied by an assumed average annual profit per ewe of NZD 40 for fine-wool and NZD 35 for other breeds based on expert opinion, the assumed proportion of live ewes in a flock with BCS ≤ 1.5 (i.e., 5% of the total flock for both breed categories based on expert opinion) and the proportion of ELISA positive ewes.

### 2.4. Data Processing and Statistical Analyses

Data on ewe mortality, necropsy, histopathology and serology were stored in an online database called IRIS maintained by the Epicentre at Massey University, New Zealand. All calculations were done separately for fine-wool and other breeds. Statistical analyses were conducted using an open source computer program R, version 3.1.3 (2015-03-09) for Windows [10]. A probability of p < 0.05 was considered statistically significant and confidence intervals were computed at 95%.

### 2.5. Economic Analysis

To estimate economic effects and benefit-cost analysis of vaccination, various cost and revenue parameters were calculated. If necessary data were not available from this study or literature, they were based on expert opinion. These parameters and assumptions of stochastic modelling of OJD economics are presented in Table 2. The vaccine efficacy estimate used in this study was from a clinical trial on self-replacing Merino farms that had >5% annual OJD-related mortalities in Australia [11]. We assumed that only female replacement lambs were vaccinated. In the calculation, 50% of other breed lambs were vaccinated while 60% of fine-wool were vaccinated because of lower reproductive rates in these breeds. Likely variable cost and revenue parameters were subjected to stochastic uncertainty (Table 2). Parameters from expert opinion that were held constant during stochastic modelling are shown in Table 3. Economic effects were estimated for a hypothetical flock of 2000 breeding ewes.

In addition to OJD-specific mortality, economic outcomes linked to OJD mortality, overall OJD-related loss at farm-level and benefit-cost of vaccination were based on stochastic simulation involving ten thousand random draws. The use of stochastic simulation accounted for uncertainties around parameters used for estimation of OJD-specific mortalities, economic outcomes linked to OJD mortality, as well as production cost and revenue figures taken from other sources mentioned above. Consequently, outputs are shown as posterior distributions.

## 3. Results

Thirteen fine-wool and four other breed properties provided ewe tallies, scanning and tailing data and submitted ewes for necropsy. Of the farms that did not provide tally records, the two fine-wool farms contributed sheep for necropsy, while the other breed farm provided serum samples for ELISA but neither tallies nor ewes for necropsy.

Average tailing percentage per ewe was 103% (range between farms 66–135%), but these estimates were highly dependent on data from fine-wool farms as only two farm-year data sets for tailing percentage were available for the other breed.

### 3.1. Ewe Mortality

The crude incidence rate of annual ewe mortality (step 1) was 7.6% (n = 109,320 ewes, 8287 deaths; CI 6.5–8.6%). No significant difference was observed between fine-wool (7.6%, CI 6.3–8.8%) and other breeds (7.6%, CI 5.2–9.9%).

In step 2, 36% (SD 14%) of all ewe deaths were ‘likely OJD related’ in fine-wool breeds compared with 21% (SD 25%) in other breeds.

Among necropsied ewes (step 3), OJD was the cause of wasting or death in 68% (CI 60–79%) of fine-wool vs. 27% (CI 4–50%) of other breeds (p < 0.001). In total, OJD was considered to be the major cause of death in 218 out of 358 necropsied ewes, which had more widespread lesions in multiple areas of the intestine including the Peyer’s patches and associated mucosa and conformed to Type 2 (n = 3 ewes) and 3a–c (n = 215 ewes) lesions described by Pérez et al. [9]. The age distribution of necropsied ewes was normal, but ewes with confirmed OJD were 0.6 years younger (p = 0.001) than those dying of other causes, after controlling for the effect of farm. The lower age of confirmed cases was independent of breed since the interaction term between breed and OJD outcome was not significant.

### 3.2. ELISA

Of the 228 ewes tested with serum ELISA, 22.3% were positive overall, with 40% (CI 18–61%; n = 101) of fine-wool and 9% (CI 0–22%; n = 127) of other breed ewes testing positive (p < 0.001).

### 3.3. Stochastic Simulation

**Figure 1:**
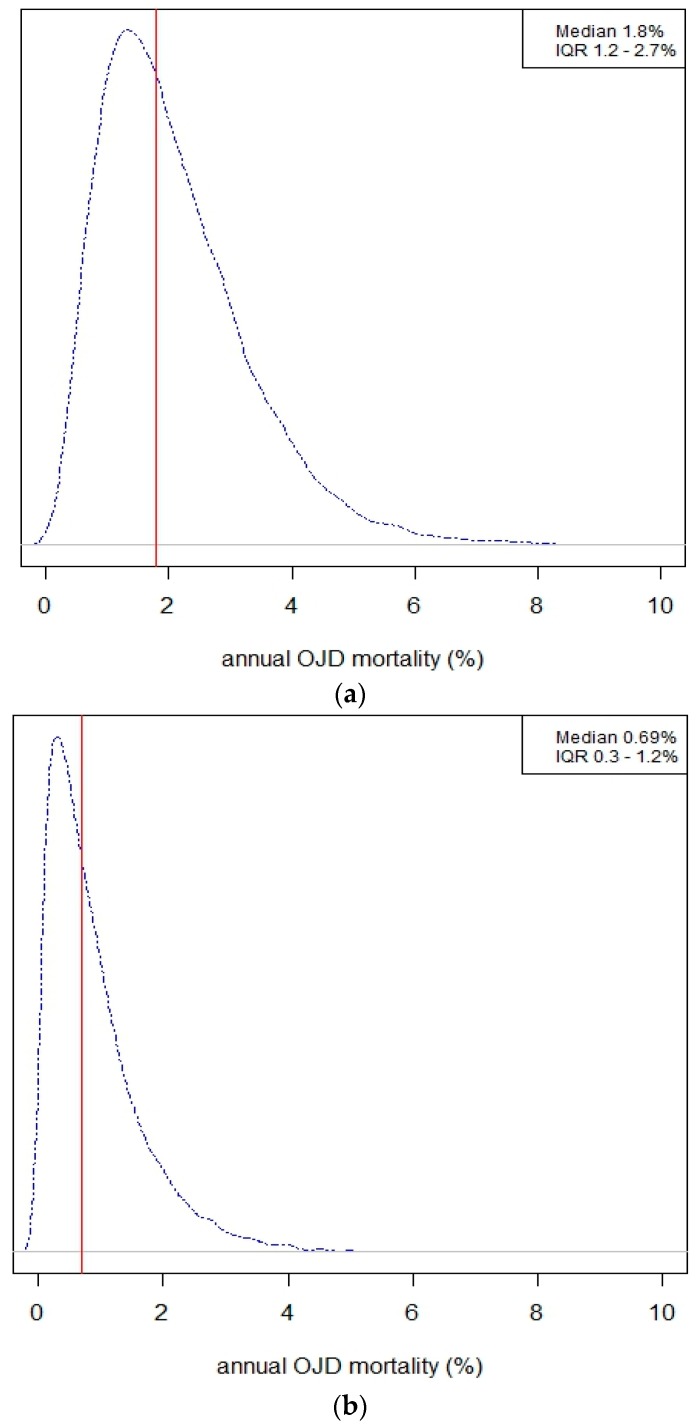
Density plots of estimated annual OJD mortality of ewes in fine-wool (**a**) and other breed (**b**) flocks and interquartile range (IQR, 25th–75th percentiles). The vertical line represents the median.

Based on the stochastic simulation, annual OJD mortality was 2.7 times as high in fine-wool (median 1.83%; IQR 1.2–2.7%) than in other breeds (median 0.68%; IQR 0.3–1.2%) (Figure 1).

**Figure 2:**
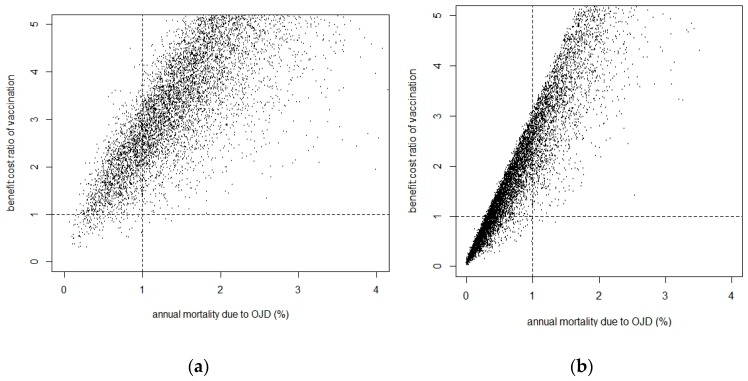
Scatter plots of correlation between annual mortality due to OJD and benefit-cost ratio of vaccination in fine wool (**a**) and other breeds (**b**). The dotted horizontal line represents the breakeven point above which vaccination is beneficial and the dotted vertical line represents the cut point of annual mortality above which most of the flocks have a benefit: cost ratio above one.

**Figure 3:**
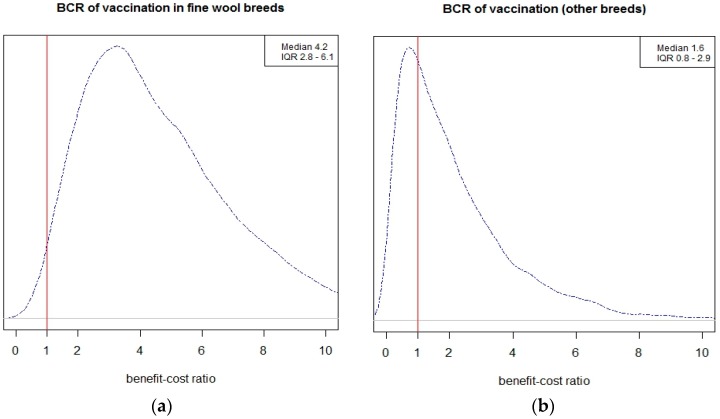
Density plots of benefit-cost ratio in fine-wool (**a**) and other breeds (**b**) and interquartile range (IQR, 25th–75th percentiles). The vertical line represents the breakeven point where benefit-cost ratio is one.

Stochastic analysis and assumptions for estimation of benefit-cost of vaccination is presented in Table 4. Annual economic cost due to OJD mortality and benefit-cost ratio of vaccination were highly correlated, with positive financial return occurring from vaccination when the annual mortality rate due to OJD was more than 1% (Figure 2). The resulting posterior distribution of the benefit-cost of OJD vaccination is illustrated in Figure 3.

For farms with fine-wool breeds, the average incidence rate of 1.8% OJD mortality per year resulted in an average return of NZD 4.2 (IQR 2.8–6.1) for NZD1 invested in vaccination. The equivalent return for other breeds, with an average of 0.68% OJD mortality, was NZD 1.6 (IQR 0.8–2.9). Returns above the breakeven point were realised in at least 95% and 65% of the simulated fine-wool and other breed flocks, respectively. Annual ewe mortality due to OJD was the most important determinant of economic return from vaccination.

Costs due to OJD-associated lamb, ewe, replacement and pre-clinical loss in the two breed categories are shown in Table 5. For farms with the average incidence rate of OJD mortality per year the annual loss for a farm with 2000 fine-wool ewes would be NZD 13,100 (IQR 8900–18,600) and NZD 4300 (IQR 2200–7600) for other breeds. Differences in annual economic loss between the two breed categories were attributable to breed-specific differences in reproductive performance and value of animal for sale.

## 4. Discussion

In this study mortality rate in fine-wool ewes was not different from that in other breeds, but the rate attributable to OJD was more than twice as high in fine-wool breeds, which suggested higher susceptibility of the latter category ewes to clinical OJD. This was not unexpected as the majority of fine-wool farms in this study were Merino farms and earlier studies from New Zealand and overseas have reported Merino breeds being more susceptible to mortality due to OJD than other breeds [7,12]. The stochastic analysis showed it would be more economical to vaccinate replacement lambs if the pre-vaccination annual ewe mortality s more than 1%.

Large between-farm variation of OJD-specific mortality was observed for fine-wool breeds, though sample size for other breeds was limited and there was considerable uncertainty about estimated total mortality. Variable OJD mortality indicates that intervention is not warranted on every fine-wool farm. Hence, farm-specific OJD incidence should be objectively and reliably established before interventions such as vaccination are implemented, particularly when economic return is an imperative. The relatively small variation in OJD mortality for other breeds was possibly a chance effect due to few farm-years data, hence more information about other breeds is required before strong inferences can be drawn.

Ewe death rate due to OJD was estimated indirectly based on three mortality categories. Ewes that farmers presented as terminal culls for necropsy after a chronic condition were deemed ‘possibly OJD-related culls’. Other categories were ewes in extremely poor condition killed for ‘dog tucker’, and those found dead on pasture were also considered possible OJD cases. It was assumed that the latter categories at least partially represented ewes that a farmer would otherwise have presented for necropsy. While this was subjective and therefore not always correct, it was considered to be a reasonable interpretation within the logistic limits of the study. The proportion of ‘possibly OJD-related culls’ was considered a reasonable means of establishing that ewes subjected to necropsy represented all ewes that died or were culled due to terminal conditions.

Ewes classified as OJD-affected were those with low BCS but not marked for culling. They comprised a small proportion of any flock. For economic analyses it was deemed appropriate to consider that they were expected to have a shorter life-span than ewes with higher BCS. This group of ewes therefore contributed to the overall OJD-specific loss tallies, in conjunction with culled/dead ewes.

Sensitivity of the serum ELISA test is higher in animals showing symptoms of disease than in latently infected animals [13]. While specificity of this assay is high at approximately 99% [14], thereby limiting false positive misclassification of ewes clinically affected by OJD, its sensitivity may range from 22–46% in latently infected clinically healthy sheep [14]. This low probability of unaffected ewes testing positive by ELISA was helpful for the economic analysis in that most infected animals that were not yet pre-clinical OJD cases would be excluded. This supports that the ELISA was a suitable test for estimating the proportion of OJD-affected live ewes.

To estimate the cost-effectiveness of vaccination, we used data on vaccine efficacy reported in the literature [11]. Gudair^TM^ (Map 316F strain, Zoetis, Australia), which is registered for use in sheep in New Zealand, was evaluated by clinical trial on three Merino farms in Australia by Reddacliff et al. [11] who reported 90% reduction in OJD mortality after one year compared with controls. In a clinical trial of young deer in New Zealand, vaccine efficacy of 60% was reported in terms of decreasing incidence of clinical disease [15]. To account for the possible variation in vaccine efficacy between farms, vaccine efficacy along with other variable parameters were subjected to stochastic simulation.

In this simulation, we estimate the benefit-cost analysis of vaccination at a time when OJD had reached a state of equilibrium (constant prevalence of infection). It is therefore a snap-shot and ignores infection dynamics over time, and thus the time during which replacement lambs were vaccinated but no benefits were received due to ongoing mortality of adult ewes. Hence, based on this study we can only get an estimate of the economic return after the full effect of vaccination has been realised. Accounting for the real and discounted return over time would reduce the benefit-cost because a farmer would have to invest in vaccinating the flock for number of years before OJD was sufficiently controlled to prevent most or all of the economic loss attributable to the disease.

Literature review suggests that as annual mortality due to OJD increases, the time required for obtaining a positive return on vaccination investment decreases. Nevertheless, direct comparison between studies is complicated due to differences in model types, definition and number of model parameters as well as sheep production systems in the country of study.

A recent study modelling OJD in New Zealand Romney flocks [16], reported mixed-aged ewe flocks with 1% OJD mortality incidence per year might reach the breakeven (when the annual net profit of vaccination becomes positive), after five years. However, depending on the annual incidence of OJD, it might take several more years for the cumulative benefit of vaccination to be higher than the cumulative cost of vaccination, for example 30, 15 and 10 years for 0.97%, 1.1% OJD and 1.27% OJD incidence, respectively.

Bush et al. [17] modelled the benefit of OJD vaccination over 20 years in Australian Merino flocks with different initial levels of annual OJD mortality. They suggested in most cases the breakeven point may be reached in four years if the initial disease mortality was high (>7%), 5 years if the mortality was medium (3–7%), or 10 years if the mortality was lower (<3%).

Although post-vaccination lesions were reported to persist for up to four years, they did not result in carcass losses or downgrading [18]. Hence, no cost was attributed to trimming or downgrading of carcasses during processing in our stochastic model. Regulations governing the management of OJD vaccinated stock in New Zealand have recently been changed such that trimming or detaining of carcasses is no longer prescribed.

The large between-farm variation for most input parameters including mortality and replacement rates and estimated vaccine cost-effectiveness generated by our simulation will not hold for every farm. Hence many lamb-producing farms in particular (i.e., ‘other breeds’) would likely not profit from vaccination. Our economic analysis indicated that vaccination may be advisable and cost effective in farms where OJD mortality was higher than 1%. Brett (1998) suggested a similar threshold reporting that it was economically beneficial to vaccinate if the clinical incidence of OJD was at least 1% in breeding ewes provided that there was no deduction in monetary value of a carcass value due to vaccination.

In this study, we used stochastic simulation. In deterministic calculations all parameters have point average values that are assumed to be constant. Our estimation of OJD-specific mortality and economic outcomes were based on several biological parameters. Inherently, such parameters are subject to biological and chance variation. Stochastic simulation took this into account by repeatedly sampling from parameter distributions thousands of times. The resulting posterior distribution of the benefit–cost- of OJD vaccination therefore considered the biological variability of all input parameters to provide greater insight into the likelihood for a farm to achieve a positive financial return from vaccination, as illustrated in Figure 2.

In this study we used 20 farms known to have OJD and with owners or managers willing and able to participate and record the data required by the study design. This is therefore a potentially biased subset of the population, hence any inference beyond the farms and flocks included in the study may or may not be representative the population. By necessity, this study design was pragmatic since selecting a truly random subset of sheep farms in the population is not feasible when reliance on farmer choice to participate is an imperative. Nevertheless, the enrolled farms can be considered typical of fine-wool farms that view OJD as a problem. However, only a few farms with other breeds were included in the field studies, thus if anything, they might represent lamb producing farms with a comparatively high OJD incidence.

This study focused on economic loss due to both clinical and preclinical Johne’s disease in ewes. Since infection with Map does not always progress to clinical disease in all sheep [19], it might be worthwhile to investigate the economic impact of subclinical paratuberculosis on ewe production traits such as growth, lamb production and wool quantity or quality. The economic impact of sub-clinical paratuberculosis in sheep is poorly documented and to the best of our knowledge only one previous study conducted in New Zealand investigated the effect of subclinical paratuberculosis on productivity in sheep [20]. That study found subclinical disease may not lead to significant production loss in sheep, but the results were based on study of only two farms. Thus, a longitudinal study incorporating a larger number of farms might help to better understand the impact of sub-clinical disease in sheep and evaluation of the economic effects.

## 5. Conclusions

This study presents estimates of mortality rates for OJD, its economic impact, and vaccination benefit-cost. It confirms that OJD mortality rate is higher in fine-wool breeds such as Merino than in meat breeds such as Romney. The incidence of OJD mortality in a flock was the most important determinant of economic cost and therefore benefit-cost of vaccination. Large variation was observed between farms and on some farms between years, hence the economic return of any intervention would also vary accordingly. The dependence on the clinical OJD incidence for economic return demonstrates that farmers need to tally OJD cases on-farm to inform decisions around adoption of vaccination, regardless of breed type.

The stochastic simulation addressed the state of OJD at equilibrium. At that stage, vaccination was cost-effective when the annual incidence of OJD mortality prior to vaccination was 1% or more. For fine-wool breeds, this provided a benefit-cost ratio of four, but this required a number of years of continued vaccination.

## Figures and Tables

**Table 1:** Flock tallies of study farms by breed.

Farm	Breed	Years Monitored	Ewes Mated	Total Missing	% Ewe Mortality
Fine-Wool					
A	Corriedale	2012–2013	1040	29	2.8%
		2013–2014	1091	78	7.1%
B	Half-bred	2012–2013	2130	136	6.4%
		2013–2014	2316	154	6.6%
C	Merino	2012–2013	3106	209	6.7%
		2013–2014	3320	260	7.8%
D	Merino	2012–2013	5300	512	9.7%
		2013–2014	4631	552	11.9%
E	Merino	2012–2013	3670	135	3.7%
		2013–2014	3867	151	3.9%
F	Merino	2012–2013	3459	212	6.1%
		2013–2014	3595	177	4.9%
G	Merino	2012–2013	2763	195	7.1%
		2013–2014	2860	203	7.1%
H	Merino	2012–2013	8402	1317	15.7%
		2013–2014	7909	461	5.8%
I	Merino	2013–2014	3926	254	6.5%
J	Merino	2013–2014	4699	338	7.2%
K	Merino	2012–2013	1780	89	5%
		2013–2014	1807	77	4.3%
L	Merino	2012–2013	2590	120	4.6%
		2013–2014	2668	132	4.9%
M	Merino	2013–2014	2244	212	9.4%
Others					
N	Romney	2012–2013	2010	226	11.2%
		2313–2014	2190	73	3.3%
O	Romney	2013–2014	20,104	1498	7.5%
P	Composite	2012–2013	2064	167	8.1%
		2013–2014	2104	172	8.2%
Q	Composite	2012–2013	890	93	10.4%
		2013–2014	785	55	7%

**Table 2:** Calculated and expert opinion based parameters and assumptions used for stochastic simulation modelling of OJD economics.

Measurement	Distribution	Parameters of the Distribution		Assumptions about Parameters		
		Fine-Wool	Other Breeds	Fine-Wool	Other Breeds	Source
Annual overall mortality	beta	a = 8.717 b = 94.8221	a = 10.2378 b = 113.3121	mode = 0.076 95% sure <0.133	mode = 0.076 95% sure <0.127	calculated
OJD as suspected by farmers	beta	a = 3.9543 b = 6.2522	a = 1.4972 b = 2.8591	mode = 0.36 95% sure <0.64	mode = 0.211 95% sure <0.77	calculated
OJD confirmed by necropsy	beta	a = 4.9044 b = 2.711	a = 3.3551 b = 7.1501	mode = 0.6813 95% sure >0.3539	mode = 0.2698 95% sure <0.565	calculated
Average number of lambs docked per ewe per farm year (lambing percentage)	normal	mean = 1 sd = 0.17	mean = 1.31 sd = 0.05	Na	Na	calculated
Proportion of lambs with delayed finishing	normal	mean = 0.5 sd = 0.05	mean = 0.5 sd = 0.05	Na	Na	expert opinion
Days to finish post-weaning	normal	mean = 240 sd = 24	mean = 100 sd = 10	Na	Na	expert opinion
Death rate weaning to finishing (i.e., when lamb has reached marketable weight of 40–50 kg)	beta	a = 6.4243 b = 176.3867	a = 6.4817 b = 214.7868	mode = 0.03 95% sure <0.06	mode = 0.025 95% sure <0.05	expert opinion
Annual ewe replacement rate	beta	a = 3.8761 b = 9.6284	a = 3.8761 b = 9.6284	mode = 0.2595% sure <0.5	mode = 0.25 95% sure <0.5	expert opinion
Proportion of live ewes in low BCS (≤1.5) that tested ELISA positive	beta	a = 3.0818 b = 3.1668	a = 15.41 b = 130.70	mode = 0.39 90% sure <0.64	mode = 0.08 95% sure <0.26	calculated
Proportion of ewes with low BCS (≤1.5)	beta	a = 6.1946 b = 99.6983	a = 6.1946 b = 99.6983	mode = 0.05 95% sure <0.1	mode = 0.05 95% sure <0.1	expert opinion
Profit per ewe per year	normal	mean = 45 sd = 4.5	mean = 35 sd = 3.5	Na	Na	expert opinion
Productive years lost due to OJD	normal	mean = 0.48 sd = 0.048	mean = 0.48 sd = 0.048	Na	Na	expert opinion
Vaccine efficacy in terms of reducing mortality	beta	a = 5.3842 b = 1.4871	a = 5.3842 b = 1.4871	mode = 0.9 95% sure >0.5	mode = 0.9 95% sure >0.5	[10]

Note: Na = Not applicable; BCS = body condition score.

**Table 3:** Revenue and production cost parameters based on expert opinion. These parameters were held constant for simulation modelling of OJD economics.

Assumptions	Fine-Wool Breeds	Other Breeds
Revenue from meat per lamb sold (NZD)	90	90
Revenue from fleece per ewe (NZD)	40	17
Revenue from fleece per lamb (NZD)	40	0
Revenue from meat/salvage ewe (NZD)	50	70
Revenue from fleece/salvage ewe (NZD)	40	17
Cost of a replacement ewe (NZD)	105	105
Health and feed cost per day to finish (NZD)	0.11	0.11
Proportion of ewes with BCS < 1.5	5%	5%
Cost of vaccine per dose (NZD)	3.50	3.50
Labour cost of vaccination per ewe (NZD)	0.50	0.50
Proportion of ewe lambs born that were vaccinated	60%	50%

**Table 4:** Stochastic analysis, and assumptions used, for estimation of benefit-cost of vaccination of lambs in a hypothetical fine-wool and other breed flock of 2000 ewes, based on calculated mortality rates from this study and 90% vaccine efficacy for reducing mortality.

Measurements	Fine-Wool	Other Breeds ^1^
Cost of Vaccination		
% ewe lambs vaccinated	60%	50%
No. ewe lambs vaccinated	600	500
Total lamb vaccine cost (NZD)	2400	2000
Benefit from Vaccination		
Achievable benefit (in thousands NZD)	10.1 (6.7–14.8) ^2^	3.3 (1.6–5.9) ^2^
Benefit-cost ratio (BCR)	4.2 (2.8–6.1) ^2^	1.6 (0.83–2.9) ^2^

^1^ Due to few farms contributing data, estimates for ‘other breeds’ are farm specific, and not necessarily population average. ^2^ Figures represent median and range within parenthesis represent the interquartile range.

**Table 5:** Median (and interquartile range) annual cost of OJD for a hypothetical farm of 2000 fine-wool or other breed ewes at average annual mortality rates observed in this study.

Measurements	Fine-Wool (IQR)	Other Breeds (IQR)
Annual OJD mortality %	1.83 (1.2–2.7)	0.68 (0.33–1.2)
Ewe loss (in thousands NZD)	3.3 (2.1–4.9)	1.2 (0.6–2.1)
Lamb loss in terms of opportunity Loss due to ewes with OJD (in thousands NZD)	4.2 (2.7–6.3)	1.5 (0.7–2.7)
Ewe replacement cost (in thousands NZD)	3.8 (2.5–5.7)	1.4 (0.7–2.5)
Cost of preclinical OJD (in thousands NZD)	1.3 (0.9–2.0)	0.12 (0.08–0.18)
Total loss due to OJD (in thousands NZD)	13.1 (8.9–18.6)	4.3 (2.2–7.6)
OJD production cost per ewe (NZD)	6.5 (4.4–9.3)	2.1 (1.1–3.8)

Note: IQR interquartile range.

## Abbreviations

BCS	body condition score
CI	confidence interval
ELISA	enzyme linked immunosorbent assay
IQR	interquartile range
Map	*Mycobacterium avium paratuberculosis*
NZD	New Zealand dollars
OJD	ovine Johne’s disease
